# Supplementary material for: Exploring Helium Ions’ Potential for Post-Mastectomy Left-Sided Breast Cancer Radiotherapy
Source: Cancers (Basel). 2024 Jan 18;16(2):410. doi: 10.3390/cancers16020410 (PMC10814201; doi:10.3390/cancers16020410)
Supplement: Supplementary file 1 [file cancers-16-00410-s001.zip › cancers-2693696-supplementary.pdf]

cancers

Supplementary Materials

In this section, additional patient information's were reported.

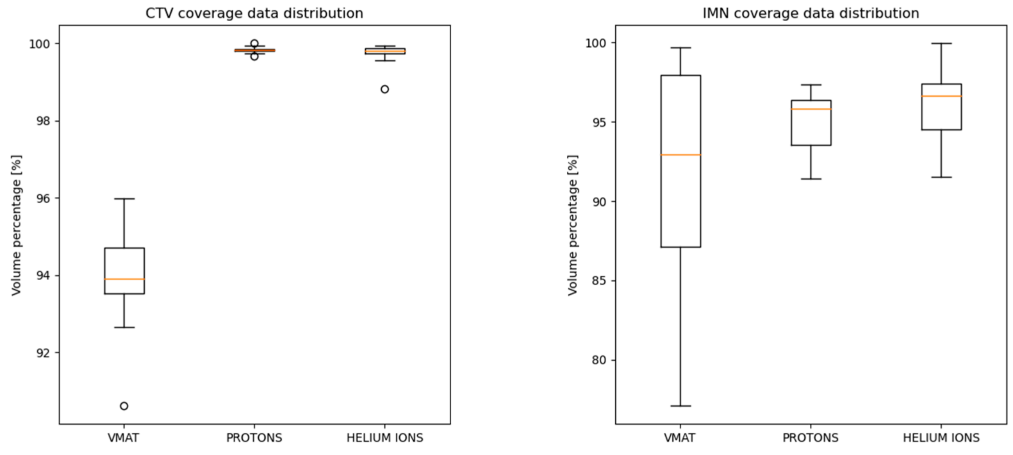

(**a**) (**b**)

Figure S1. Boxplots for (a) CTV and (b) IMN coverages in all VMAT, proton and helium plans. CTV: clinical target volume, IMN: Internal Mammary lymph Nodes, VMAT: Volumetric Modulated Arc Therapy.

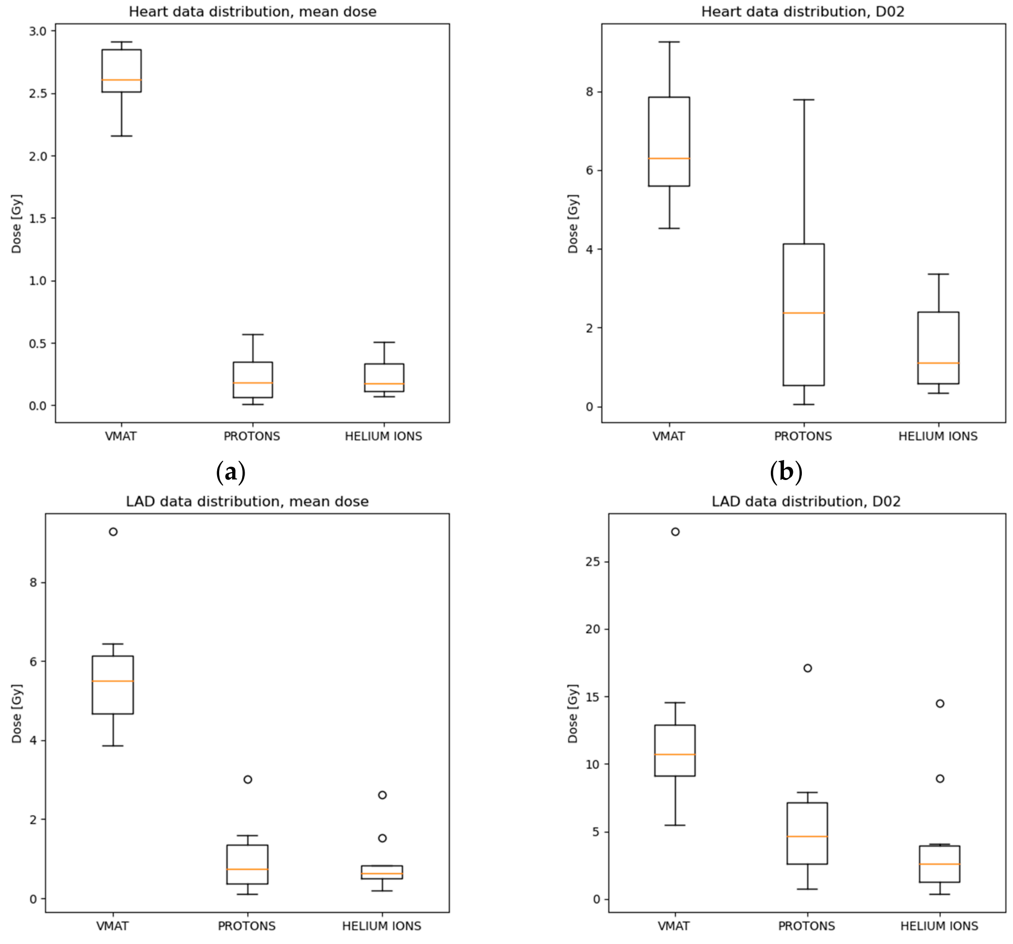

(**a**) (**b**)

(**c**) (**d**)

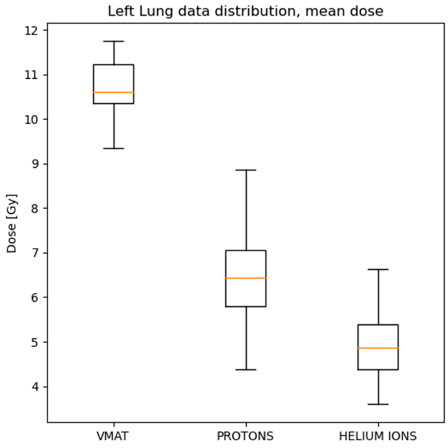

(**e**)

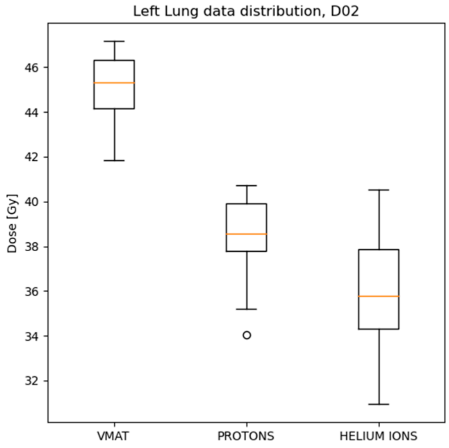

(**f**)

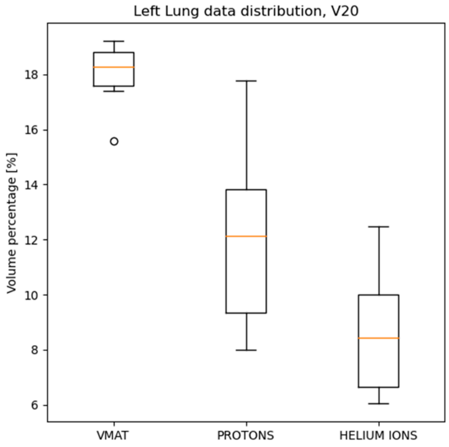

(**g**)

Figure S2. Boxplots of mean dose value and D_2 for (a)(b) heart, (c)(d) LAD and (e)(f) left lung. In (f) the V_{20Gy} percentages values distribution left lung respectively for VMAT, proton and helium plans. This box plot encloses the range of patient data distribution. CTV: clinical target volume, IMN: Internal Mammary lymph Nodes, LAD: left anterior descending artery, VMAT: Volumetric Modulated Arc Therapy.

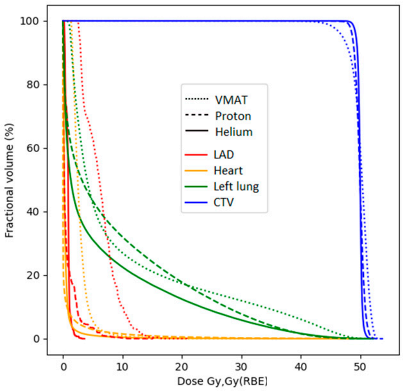

(**a**)

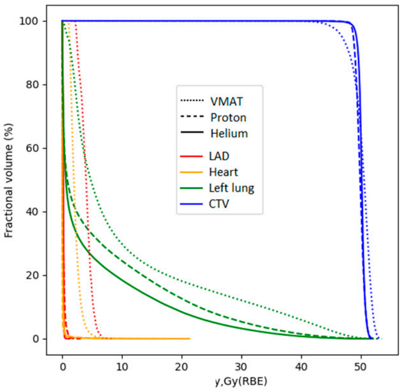

(**b**)

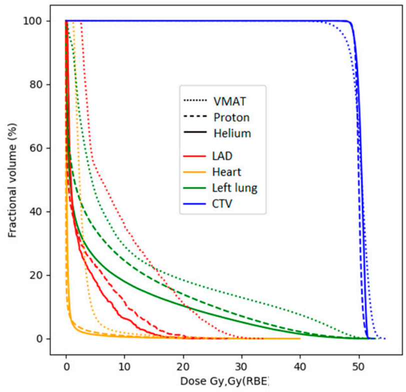
Fractional volume (%)
Dose Gy,Gy(RBE
VMAT
Proton
Helium
LAD
Heart
Left lung
CTV

(**c**)

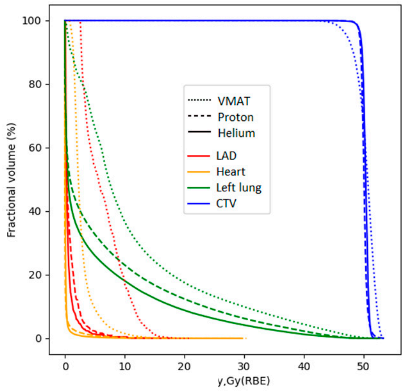
Fractional volume (%)
y,Gy(RBE)
VMAT
Proton
Helium
LAD
Heart
Left lung
CTV

(**d**)

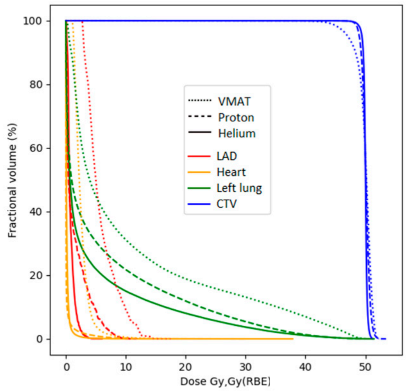
Fractional volume (%)
Dose Gy,Gy(RBE)
VMAT
Proton
Helium
LAD
Heart
Left lung
CTV

(**e**)

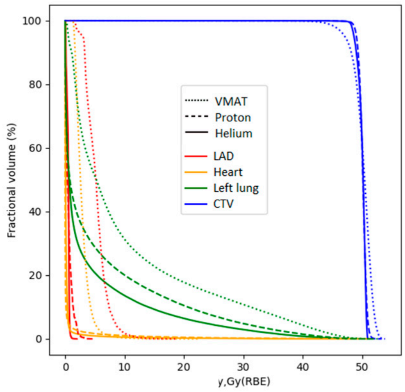
Fractional volume (%)
y,Gy(RBE)
VMAT
Proton
Helium
LAD
Heart
Left lung
CTV

(**f**)

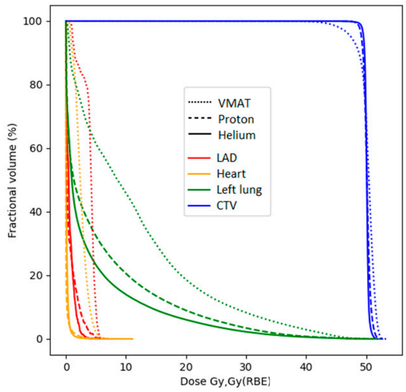
Fractional volume (%)
Dose Gy,Gy(RBE)
VMAT
Proton
Helium
LAD
Heart
Left lung
CTV

(**g**)

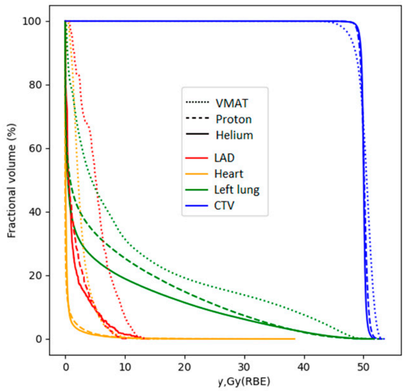
Fractional volume (%)
y,Gy(RBE)
VMAT
Proton
Helium
LAD
Heart
Left lung
CTV

(**h**)

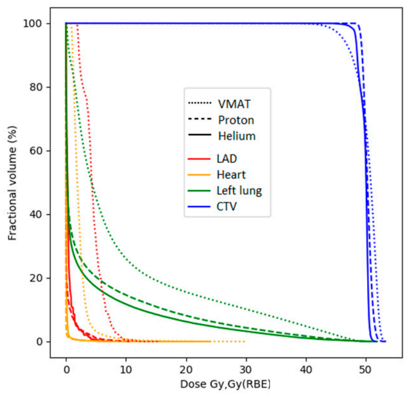

(i)

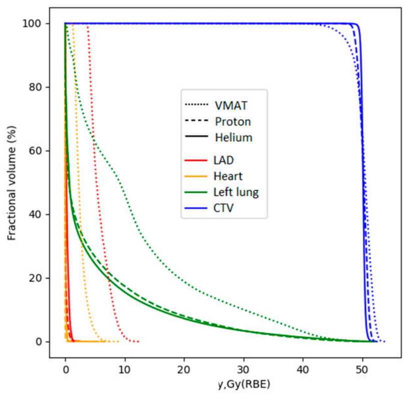

(l)

Figure S3. (a)-(l) Dose volume histograms (DVHs) for all patients. In blue the CTV coverage, in red the LAD, in green the left lung and in orange the heart. The data from the VMAT plan are represented with dotted lines, from proton plan with dashed lines and for the helium with solid lines. CTV: clinical target volume, LAD: left anterior descending artery, VMAT: volumetric modulated arc therapy**.**
